# Quality of life and abstinence in alcohol use disorders

**DOI:** 10.1192/j.eurpsy.2021.1518

**Published:** 2021-08-13

**Authors:** J. Teixeira, S. Ferreira, L. Moutinho

**Affiliations:** 1 Clínica 4 - Unidade De Alcoologia E Novas Dependências, Centro Hospitalar Psiquiátrico de Lisboa, Lisboa, Portugal; 2 Utra, CHPL, Lisboa, Portugal

**Keywords:** abstinence, quality of life, alcohol use disorders

## Abstract

**Introduction:**

The analysis of the impact of individuals’ behaviors on their health involves several variables, namely alcoholism. It is necessary to take in account that when anxiety is excessive it may be very disabling and produce many adverse effects, such as unsatisfactory work performance, anxiety disorders, depressive mood and somatic symptoms. These elements affect the Quality of Life (QOL) drastically, while social support of the patients protect QOL.

**Objectives:**

To assess the quality of life of patients with alcohol use disorders in treatment for alcohol use disorder (AUD).

**Methods:**

An exploratory, descriptive and correlational study was carried out. A sociodemographic scale was used, an instrument constructed by the authors that assesses the existence of risk behaviors and protective health behaviors, the Social Support Satisfaction Scale (ESSS), and a quality of life assessment scale (WHOQOL-Bref). Data analysis was performed using IBM SPSS 25 statistics.

**Results:**

Sample consisting of 34 patients with Alcohol Use Disorders. Abstinence time is positively correlated with QOL and negatively correlated with social support satisfaction.

**Conclusions:**

This study shows that in treatment of patients with AUD, longer abstinence times have a positive effect on QOL and overall wellbeing of patients, while being associated with a lower satisfaction with social support. Treatment Units dedicated to AUD should keep striving for maintenance of abstinence due to these positive effects.

